# Clinical, genetic and structural delineation of *RPL13*-related spondyloepimetaphyseal dysplasia suggest extra-ribosomal functions of eL13

**DOI:** 10.1038/s41525-023-00380-x

**Published:** 2023-11-22

**Authors:** Prince Jacob, Hillevi Lindelöf, Cecilie F. Rustad, Vernon Reid Sutton, Shahida Moosa, Prajna Udupa, Anna Hammarsjö, Gandham SriLakshmi Bhavani, Dominyka Batkovskyte, Kristian Tveten, Ashwin Dalal, Eva Horemuzova, Ann Nordgren, Emma Tham, Hitesh Shah, Else Merckoll, Laura Orellana, Gen Nishimura, Katta M. Girisha, Giedre Grigelioniene

**Affiliations:** 1 Department of Medical Genetics, Kasturba Medical College, Manipal, Manipal Academy of Higher Education, Manipal, India; 2 Department of Molecular Medicine and Surgery, Karolinska Institutet, Stockholm, Sweden; 3 Department of Clinical Genetics, Karolinska University Hospital, Stockholm, Sweden; 4 Department of Medial Genetics, Oslo University Hospital, Oslo, Norway; 5 Department of Molecular & Human Genetics, Baylor College of Medicine and Texas Children’s Hospital, Houston, TX USA; 6 Division of Molecular Biology and Human Genetics, Faculty of Medicine and Health Sciences, Stellenbosch University and Medical Genetics, Tygerberg Hospital, Cape Town, South Africa; 7 Department of Medical Genetics, Telemark Hospital Trust, Skien, Norway; 8 Diagnostics Division, Centre for DNA Fingerprinting & Diagnostics, Hyderabad, India; 9 Department of Clinical Genetics and Genomics, Sahlgrenska University Hospital, Gothenburg, Sweden; 10 Institute of Biomedicine, Department of Laboratory Medicine, University of Gothenburg, Gothenburg, Sweden; 11 Department of Pediatric Orthopedics, Kasturba Medical College, Manipal, Manipal Academy of Higher Education, Manipal, India; 12 Department of Radiology, Oslo University Hospital, Oslo, Norway; 13 Protein Dynamics and Mutation lab, Department of Oncology-Pathology, Karolinska Institutet, Stockholm, Sweden; 14 Department of Radiology, Musashino-Yowakai Hospital, Tokyo, Japan

**Keywords:** Genetics research, Molecular medicine, Disease genetics, Growth disorders

## Abstract

Spondyloepimetaphyseal dysplasia with severe short stature, *RPL13*-related (SEMD-RPL13), MIM#618728), is a rare autosomal dominant disorder characterized by short stature and skeletal changes such as mild spondylar and epimetaphyseal dysplasia affecting primarily the lower limbs. The genetic cause was first reported in 2019 by Le Caignec et al., and six disease-causing variants in the gene coding for a ribosomal protein, *RPL13* (NM_000977.3) have been identified to date. This study presents clinical and radiographic data from 12 affected individuals aged 2–64 years from seven unrelated families, showing highly variable manifestations. The affected individuals showed a range from mild to severe short stature, retaining the same radiographic pattern of spondylar- and epi-metaphyseal dysplasia, but with varying severity of the hip and knee deformities. Two new missense variants, c.548 G>A, p.(Arg183His) and c.569 G>T, p.(Arg190Leu), and a previously known splice variant c.477+1G>A were identified, confirming mutational clustering in a highly specific RNA binding motif. Structural analysis and interpretation of the variants’ impact on the protein suggests that disruption of extra-ribosomal functions of the protein through binding of mRNA may play a role in the skeletal phenotype of SEMD-RPL13. In addition, we present gonadal and somatic mosaicism for the condition.

## Introduction

**Fig. 1:**
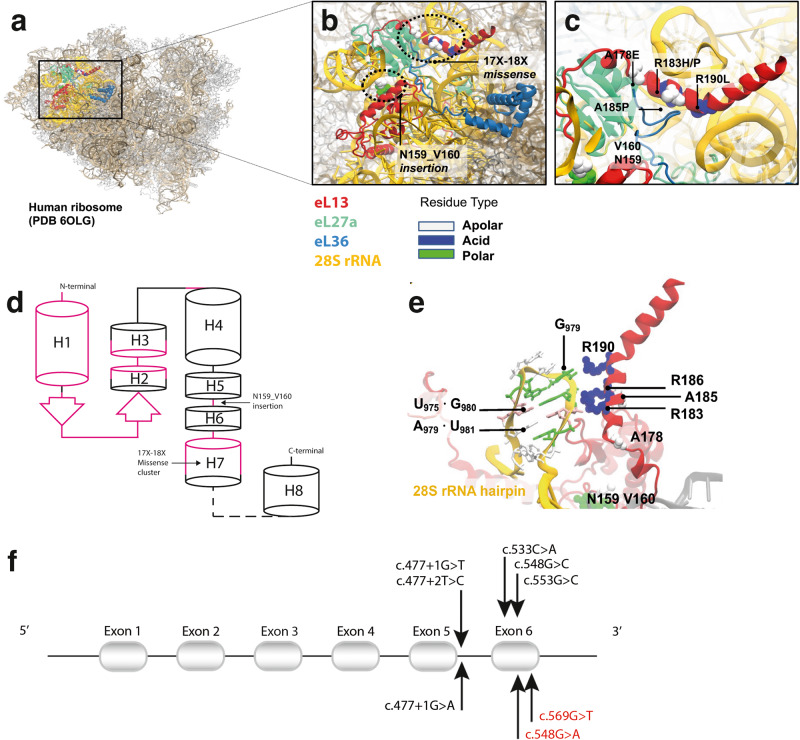
eL13 variants mapped onto a three-dimensional (3D) protein structure of eL13. **a** CryoEM structure of the *Homo sapiens* ribosome (PDB 6olg). **b** Enlarged view of eL13 (in red) shown in detail, interacting with 28S rRNA (orange) and ribosomal proteins eL36 (blue) and eL27a (green). **c** The location of the missense variants clustered on the alpha helix 7 (H7), which is a conserved region of the protein. The missense variants are either on the hydrophobic side of the helix, in contact with eL36 (A178E, A185P), or on the opposite positively charged surface, that binds the negatively charged 28S rRNA (R183H/P, R190L). The eL13 insertion point is located near the variable linker connecting helices 5 and 6. Substitutions of hydrophobic residues to proline or glutamic acid, would break the helix or introduce repulsive negative charges, while those of positively charged arginines would directly disrupt RNA binding. **d** Schematic view of eL13 secondary structure topology, indicating the location of the insertion and missense variants. The RNA binding regions across eL13 are shown in pink and eL13 28S rRNA binding is mediated by H1, beta-hairpin B1 and H7. Pathogenic eL13 variants cluster on helix 7 (H7), while the insertion targets the variable linker region connecting helix 5 and 6 (H5-H6). **e** Close-up view of the RNA-binding motif mutated in SEMD-RPL13. 28S rRNA adopts a dsRNA hairpin structure, stabilized by a wobble G.U base pair (U975.G980)^27^, recognized by eL13 R183-R185 arginine-fork^26^, that strongly binds to the nucleic phosphate backbone. The hairpin is further recognized by R190 binding to unpaired G979. Note the sharp bend in the helix at the interaction loci mediated by G·U wobble pair. **f** Schematic representation of the previously reported *RPL13* (NM_000977.3) variants (in black) along with the three disease-causing variants identified in this study (lower part). Previously unreported variants identified in this study are depicted in red.

Spondyloepimetaphyseal dysplasia (SEMD) is a group of skeletal dysplasias characterized by disproportionate short stature and varying degrees of vertebral, epiphyseal, and metaphyseal abnormalities^1–3^. The nosology of genetic skeletal disorders 2023 revision, recognize more than 20 forms of SEMDs^4^. Spondyloepimetaphyseal dysplasia with severe short stature, *RPL13*-related, (SEMD-RPL13, MIM#618728), is a recently identified form that was first reported by Isidor et al. in 2013^5^ after observing two affected children with normal birth length and early onset postnatal growth deficiency, severe short stature and *genu varum*. The radiographic features included severe epimetaphyseal changes in lower limbs, mild platyspondyly and bowed femora. Isidor et al. proposed a novel form of spondyloepimetaphyseal dysplasia with an unknown genetic etiology. Le Caignec et al. identified disease-causing variants in *RPL13*^6^ in 2019, which encodes a ribosomal protein part of the 60S subunit, eL13. Ribosomes are large ribonucleoprotein complexes composed of two subunits, a large and a small, made of approximately 80 proteins together with four ribosomal RNA molecules^7^. A schematic eL13 secondary structure is depicted in Fig. 1. Cryo-electron microscopy of human ribosomes has determined up to 36 structures for eL13, revealing two loosely packed and conserved RNA-binding regions connected by a conserved helix and an extended and variable loop that weakly interacts with RNA (Fig. S1). The N-terminal region (residues 1–100) comprises the conserved helix H1, followed by a short beta-hairpin and helices H2 and H3, which extensively bind 28S rRNA. The mid-region (residues 106–175) folds into the conserved helix (H4) and comprises a long, variable, and disordered loop that, at its C-terminus, weakly binds 28S rRNA expansion segment ES7L. Finally, the C-terminal region (residues 175–211) comprises the conserved helix H7, which extensively interacts with 28S rRNA expansion segment ES9L and, to a lesser extent, 5.8S rRNA^8^.

SEMD-RPL13 is classified as a ribosomopathy, a heterogenous group of disorders caused by abnormal ribosomal biogenesis, resulting from variants in genes coding for ribosomal proteins or those involved in ribosome biogenesis^6,9^. Ribosomopathies are highly diverse in their clinical manifestations and tissue specificity, and typically possess defects in the hematopoietic and skeletal systems^10–15^. Individuals with identical genetic aberrations in *RPL13* may exhibit varying degrees of disease severity, both within and between families, or show no manifestations at all. However, no extraskeletal symptoms, such as hematological or immunological abnormalities, have been observed in patients with SEMD-RPL13^5,6,16,17^.

A recent classification system identifies 19 distinct types of ribosomopathies categorized as pure, mixed, and acquired groups^18^. Among them, cartilage hair hypoplasia (CHH)-anauxetic dysplasia spectrum are classified as skeletal dysplasias. Ribosome synthesis is a complex process occurring in the nucleolus and cytoplasm, requiring coordination between ribosomal proteins, processing factors, and all three RNA polymerases^19,20^. Recent studies suggest that many ribosomal proteins, including eL13, possess essential functions beyond their role as structural components of the ribosome, such as binding to RNA to regulate the translation of specific mRNAs by binding to their 5’- and 3’ UTRs^21–23^.

In this report, we describe the clinical- and radiological features of 12 patients from seven unrelated families with SEMD-RPL13, providing further insight into the phenotypic spectrum of the disease. We report three disease-causing variants, two previously unreported missense variants c.548G>A, p.(Arg183His), c.569 G>T, p.(Arg190Leu) and one known splice variant c.477+1G>A, in *RPL13* (NM_000977.3). In addition, we present gonadal mosaicism for the condition in two siblings with the same disease-causing variant, which is absent in both parents’ blood DNA, and describe a case of transmission from an unaffected parent with somatic mosaicism to a child. Finally, we refine our understanding of this ultrarare disease entity by summarizing our patients’ clinical and molecular profiles alongside those previously reported with SEMD-RPL13. Our structural analysis and interpretation of the variants’ impact on eL13 reveals a precise mutational clustering in a highly specific RNA binding motif, suggesting that extra-ribosomal functions of the protein, through binding to NF-κB mRNA, may play a role in mediating the skeletal phenotype of SEMD-RPL13.

## Results

### Clinical and radiographic findings

**Fig. 2:**
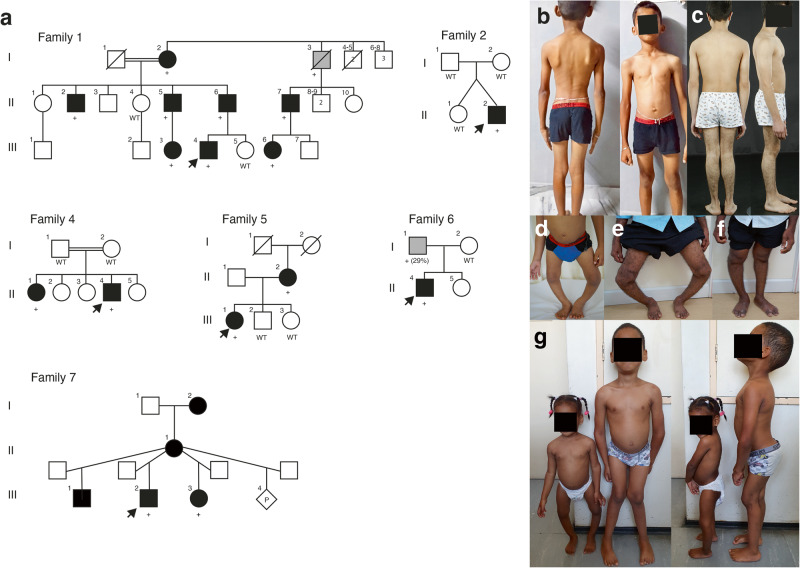
Pedigrees and clinical pictures of affected individuals. **a** Pedigrees of the families. In families 1, 2, and 5, the affected individuals (filled symbols and indicated with +) were heterozygous for c.548G>A in *RPL13*. Individuals marked with WT were tested and showed the wild-type allele. In family 4, the affected individuals were heterozygous for c.569G>A. Note that family 4 has two affected siblings, but parents were not carriers of the variant in blood DNA, suggesting parental gonadal mosaicism. In family 6 and 7, the affected individuals marked with + were heterozygous for the c.477+1G>A variant. Note that in family 6, gray color indicates mosaicism in blood DNA for the variant. In family 7, the older half-brother, mother, and maternal grandmother had suggestive clinical features but variant testing is pending. The pedigree of individual 3 is not included in this article as her family members did not agree for its publication. **b** Clinical pictures of proband 2-II:2, nine years. **c** Proband 4-II:4, 15 years. **d** Proband 1-III:4, three years, and his father 1-II:6 (**e**) and uncle 1-II:5 (**f**). **g** Half-siblings from family 7: aged 6 years and nine months and 2 years and 7 months, respectively. Note the variable severity of limb deformities. Written consent was obtained for publication of the photographs.

**Fig. 3:**
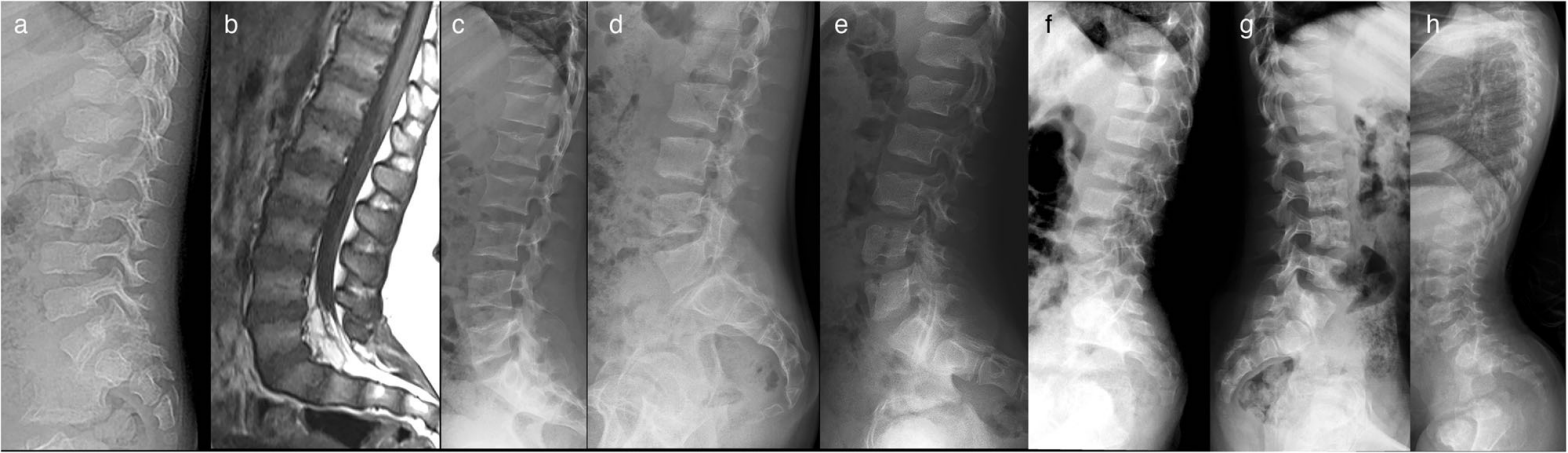
Lateral radiographs and sagittal MRI of the lumbar spine. **a** Radiograph of Patient 1-III:4 at age 3 years showing mild platyspondyly. **b** MRI of Patient 3 at age 7 years showing mild platyspondyly with irregular end plates and lumbosacral lordosis. **c** Radiograph of Patient 4-II:1 at 18 years of age showing mild platyspondyly with endplate modification. **d** Radiograph of Patient 4-II:4 at 15 years of age showing mild modification of vertebral end plates. Platyspondyly is not seen. **e** Radiograph of Patient 5-III:1 at age 7 years showing mild platyspondyly with severe lumbosacral lordosis and irregular end plates, and short neural arches of the lower lumbar spine and spinal canal stenosis. **f** Radiograph of Patient 6-II:1 at 7 years old, showing subtle irregularities of the vertebral end plates. **g** Radiograph of Patient 7-III:2 at 8 years showing vertebral bodies with central notches and anterior ossification defects. **h** Radiograph of Patient 7-III:3 at 4 years showing increased lumbosacral lordosis and vertebral bodies with anterior ossification defects and irregular end plates.

**Fig. 4:**
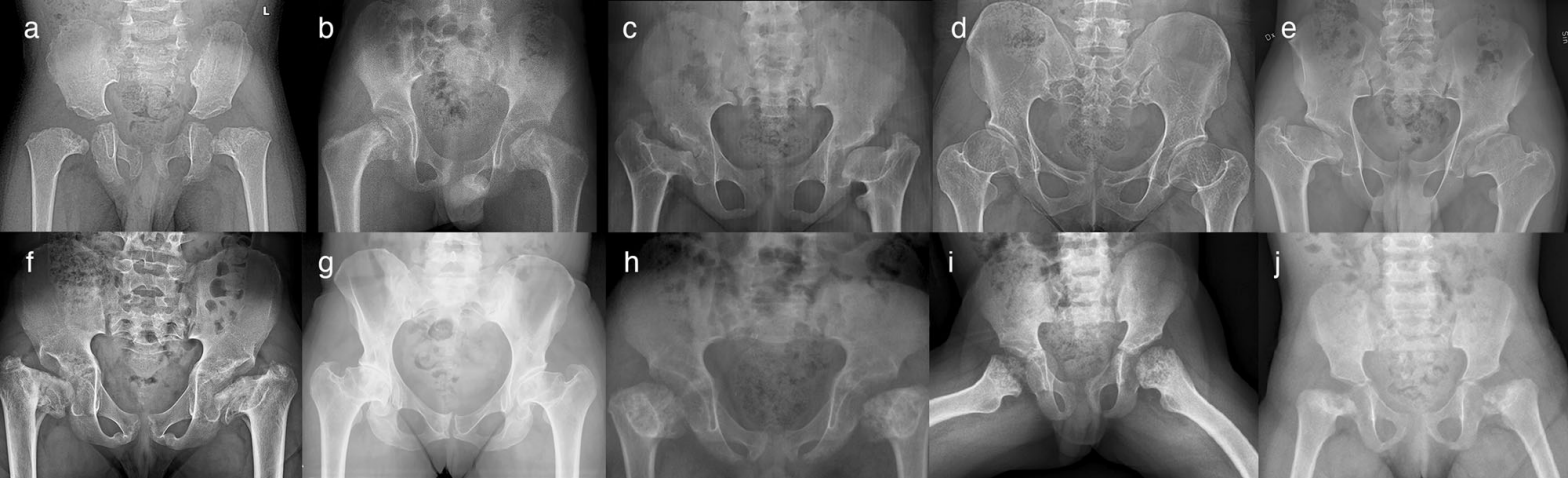
Radiographs of the pelvis. **a** Patient 1-III:4, radiograph at 3 years; showing unossified capital femoral epiphyses, and metaphyseal irregularities of the proximal femora with short femoral necks and *coxa vara*, and irregular acetabula. **b** Patient 2-II:2, radiograph at 9 years; showing flat, irregular capital femoral epiphyses, metaphyseal dysplasia of the proximal femora with short necks and *coxa vara*, and normal acetabula. **c** Patient 3, radiograph at 11 years; showing short femoral neck, severely flat capital femoral epiphyses, severe *coxa vara* and shallow acetabula, together with high-rising greater trochanters. **d** Patient 4-II:1, radiograph at 25 years; showing short femoral necks with mild *coxa vara*. The capital femoral epiphyses are not deformed, while the joint spaces are narrow. **e** Patient 4-II:4, radiograph at 15 years; showing severely flat capital femoral epiphyses and short femoral necks with mild *coxa vara* and high-rising greater trochanters. **f** Patient 5-III:1, radiograph at 10 years; showing short femoral neck, severely flat capital femoral epiphyses, severe *coxa vara*, and shallow acetabula. **g** Patient 5-II:2 the radiograph at 42 years showing flat capital femoral epiphyses, short femoral necks with mild *coxa vara* and high-rising greater trochanters, and narrow joint spaces. **h** Patient 6-II:1, radiograph at 7 years; showing short femoral necks, very small and flat capital femoral epiphyses, metaphyseal irregularities of the proximal femora, and severe *coxa vara*. **i** Patient 7-III:2, radiograph at 6 years 10 months; showing short femoral necks, flat capital femoral epiphysis of the left and delayed capital femoral epiphyseal ossification of the right, and severe metaphyseal irregularities of the proximal femora. **j** Patient 7-III:3, radiograph at 2 years and 3 months; showing short femoral necks, delayed ossification of both capital femoral epiphyses and severe metaphyseal irregularities of the proximal femora.

**Fig. 5:**
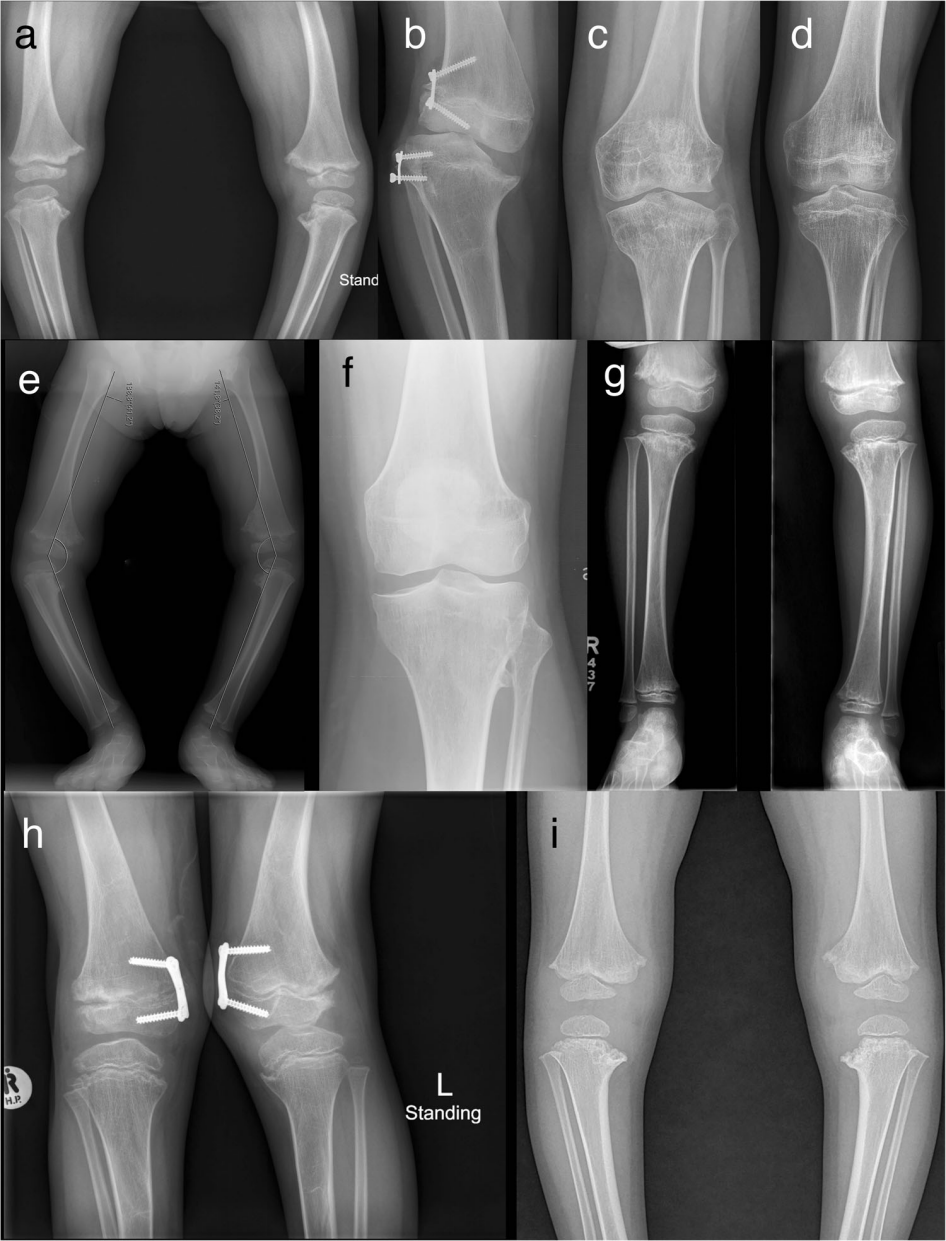
Knee radiographs. **a** Patient 1-III:4, radiograph at age 3 years; showing irregular metaphyses and small epiphyses of the distal femora and proximal tibiae with mild *genu varum*. **b** Patient 3, radiograph at age 11 years; showing *genu varum* and defective ossification of the medial aspect of the proximal tibial epiphysis. 8-plate guided growth was inserted to restore the knee deformity. **c** Patient 4-II:1 radiograph at age 18 years; showing mild epiphyseal dysplasia. **d** Patient 4-II:4, radiograph at age 15 years; showing mild epiphyseal dysplasia. **e** Patient 5-III:1, radiograph at age 3 years; showing small epiphyses and metaphyseal irregularities of the distal femora and proximal tibiae with bilateral *genu varum*. **f** Patient 5-II:2, radiograph at age 42 years; showing premature degenerative joint disease. **g** Patient 6-II:1, radiograph at 7 years; showing metaphyseal changes of the knees and mild *genu varum*. **h** Patient 7-III:2, radiograph at 7 years; showing mild metaphyseal changes and mildly flat epiphyses of the knees, *genu valgum*. 8-plate guided growth surgery partially restored severe *genu valgum*. **i** Patient 7-III:3, radiograph at 2 years; showing mild metaphyseal changes, mildly flat epiphyses of the knees and *genu varum*.

The most common features were mild to moderate short stature, delayed carpal bone ossification and epimetaphyseal dysplasia in the lower limbs. Height ranged from −5,8 to −1,2 SDS. A summary of the clinical observations can be found in Table 1 and Fig. 2, while the radiological findings are summarized in Tables S2, 3, Figs. 3–5 and Figure S2.

**Table 1:** Summary of clinical features and RPL13 variants.

Family	1			2	3	4		5		6	7	
Individual	1-III:4	1-II:6	1-II:5	2-II:2	3	4-II:1	4-II:4	5-III:1	5-II:2	6-II:1	7-III:2	7-III:3
Age (years)	3	41	35	9	20	31	24	25	64	7	6	2
Sex	M	M	M	M	F	F	M	F	F	M	M	F
Origin	Asian Indian	Asian Indian	Asian Indian	Asian Indian	Brazilian/Mixed	Turkish	Turkish	Northen European	Northen European	Hispanic	South African	South African
Height (SDS^a^)	−3.5	−5.0	−5.8	−0.6	−3.9	−1.2	−2.5	−3.1	−1.2	−2.4	−3.2	−3.2
Chest anomalies	*Pectus carinatum*, mild	–	–	*Pectus excavatum*, mild	–	–	*Pectus excavatum*	*Pectus excavatum*, mild	–	–	–	–
Scoliosis	–	NA	NA	–	–	–	+, mild	–	–	–	–	–
*Coxa vara*	+	NA	NA	+	+, severe	+, mild	+, mild	+	+, mild	+ mild	–	–
*Genu varum*	+	+	+, unilateral	–	+	–	–	+	+^b^	–	–	+
Patellar luxation	–	–	–	–	–	+	–	+	–	–	–	–
Joint hyper laxity	+	+	+	–	–	–	–	–	–	++	+	+
Other	–	–	–	–	–	–	*pes planus*	GH treated	hip replacement surgery, arthrosis	mild *genu* v*algum, pes planus*	*genu valgum, pes planus*	*pes planus*
Variants in *RPL13* (NM_000977.3)	c.548G>A	c.548G>A	c.548G>A	c.548G>A	c.569G>T	c.569G> T	c.569G>T	c.548G>A	c.548G>A	c.477+1G>A	c.477+1G >A	c.477+1G>A
Protein change	R183H	R183H	R183H	R183H	R190L	R190L	R190L	R183H	R183H	N159V160ins18	N159V160ins18	N159V160ins18

Summary of clinical features and RPL13 (NM_000977.3) variants.

*NA* not available, *M* male, *F* female, + feature present, – feature is absent, *GH* growth hormone.

^a^Patient height SDS was calculated by WHO child growth standards (http://www.who.int/childgrowth/standards/en/).

^b^Less prominent with age.

### Family 1

Family 1 had eight affected individuals (Fig. 2a, d–f). Proband I-III:4 born at full term with a normal perinatal history, presented with short stature and bilateral progressive *genu varum* at the age of 3 years (Fig. 2d). Radiographs showed unossified carpal bones, mild platyspondyly, *coxa vara*, and epimetaphyseal changes of the hips and lower extremities. The proband’s father (II:6) had severe *genu varum* at age 41 years (Fig. 2e), while the paternal uncle (II:5) had unilateral *genu varum* at 35 years (Fig. 2f). Individuals I:2, II:5, II:7, III:3 and III:6 all had *genu varum*, but further clinical data was not available. Individual I:3 was reportedly healthy without skeletal manifestations, and his height was 162 cm (−2.0 SDS). However, radiographic evaluation was not possible. All affected members in this family were heterozygous for a missense variant in *RPL13*, NM_000977.3: c.548G>A, p.(Arg183His).

### Family 2

Family 2 had one affected individual (2-II:2) (Fig. 2a, b). He was born to a non-consanguineous couple and had an unaffected twin sister. At 9 years old, he experienced walking difficulties, pain in his left leg, and general muscle weakness. Radiographs showed delayed carpal ossification, thoracic kyphosis, irregular vertebral endplates, mild platyspondyly, *coxa vara*, and epimetaphyseal changes of the hips and lower extremities. The patient had a de novo heterozygous missense variant in *RPL13*, NM_000977.3: c.548G>A, p.(Arg183His).

### Family 3

The proband in family 3 is a 20-year-old female, born to non-consanguineous parents after an uneventful pregnancy. She presented with bowed legs at an early age, clinically visible as *genu varum* from around five months of age. She underwent several surgeries on both lower extremities during childhood including 8-plate guided growth treatment (Fig. 5b). She had short stature, and radiographs showed small scaphoid and lunate, irregular vertebral endplates, mild platyspondyly, and severe *coxa vara*, as well as epimetaphyseal changes of the hips and lower extremities. She was heterozygous for *RPL13* variant, NM_000977.3: c.569G>T, p.(Arg190Leu).

### Family 4

Family 4 had two affected siblings, born to healthy first cousins of Turkish origin. The proband (4-II:4, Fig. 2a, c), a 24-year-old male and his 31-year-old sister (4-II:1) were born after normal term pregnancies with reportedly normal birth length and weights. The proband’s clinical manifestations were short stature, *pectus excavatum*, *pes planus*, and mild scoliosis. He complained of painful ankles and knees since the age of four, which limited his mobility and participation in sports activities. Skeletal examination showed irregular vertebral endplates, mild epimetaphyseal dysplasia of the knees, as well as severely flat capital femoral epiphyses and short femoral necks with mild *coxa vara* and high-rising greater trochanters.

His sister (4-II:1) had reoccurring patellar dislocation in the left knee, which required patellar stabilization surgery at eight years. She experienced pain in the knees, legs, and lower back. Radiographs showed mild platyspondyly, irregular vertebral endplates, mild lumbosacral lordosis, short femoral necks with mild *coxa vara* and mild epiphyseal dysplasia of the knees. She has a healthy three-year-old daughter, delivered by cesarean section.

Both siblings were heterozygous for a missense variant, *RPL13*, NM_000977.3:c.569G>T, p.(Arg190Leu). This variant was absent in both parents’ blood DNA, suggesting gonadal and/or somatic mosaicism in one of the parents. Parenthood was confirmed by kinship estimates from WGS using single nucleotide polymorphism data. Given that joint dislocations have not been previously reported in SEMD-RPL13, we conducted an analysis of both single nucleotide and structural variants for all known skeletal dysplasia genes according to Genomics England PanelApp gene list. Our investigation did not reveal any other potential disease candidate variants.

### Family 5

The proband in family 5 (5-III:1) was a 24-year-old female born to non-consanguineous parents of Swedish/Finnish origin at full term. She had severe bilateral *genu varum* at birth and developed short stature postnatally. Radiographs showed delayed carpal bone ossification, mild platyspondyly with severe lumbosacral lordosis, *coxa vara*, and epimetaphyseal changes of the hips and lower extremities. She was treated with recombinant growth hormone (GH) from ages five to 15 years with a dosage of 0.06 mg/kg per day and IGF-1 levels around 400μg/L. Her initial basal serum IGF-1 level was 58 μg/L (normal range for age and sex 40–310 μg/L) and her initial height was −3.1 SDS. While her height improved during the first year of treatment, to −2.25 SDS, her final height was −3.1 SDS, indicating that GH treatment probably had no significant effect on the final height.

Her mother (5-II:2), a 64-year-old female of Swedish origin, had milder skeletal abnormalities than her daughter, including mild short stature (height 155.6 cm, −1.2 SDS) and *genu varum* that became less prominent with age. At 56 years, she underwent hip replacement surgery due to degenerative joint disease. Her radiographs showed mild platyspondyly with irregular endplates, flat capital femoral epiphyses and short femoral necks with mild *coxa vara* and high-rising greater trochanters, narrow joint spaces of the hip and degenerative joint disease in the knee. At the age of 63, she was diagnosed with rectal cancer. Both individuals were heterozygous for a missense variant, *RPL13*, NM_000977.3: c.548G>A, p.(Arg183His).

### Family 6

Patient 6-II:1 was born to healthy non-consanguineous parents of Hispanic origin. In the first year of life there was a noticeable growth deceleration, and during the initial evaluation at 1.5 years of age, radiographs revealed the presence of spondyloepimetaphyseal dysplasia. Both large and small joints were observed to have joint laxity. Subsequent radiographs at 6.5 years showed normal carpal bone ossification, mildly irregular vertebral endplates, flattening of the capital femoral epiphyses with *coxa vara* and epimetaphyseal changes of the lower extremities. The patient was heterozygous for a splice variant in *RPL13*, NM_000977.3: c.477+1G>A, while his asymptomatic father was found to carry the variant in 29% of his alleles in peripheral blood DNA, consistent with a somatic mosaicism.

### Family 7

Family 7 has two affected maternal half-siblings born at term after normal pregnancies. At age 6 and 2 years, respectively, they presented with progressive short stature and lower limb deformities (Fig. 2g). The older brother (III:2) had *genu valgum*, and the younger sister (III:3) had *genu varum* and *coxa vara* (Fig. 5h, i). Both siblings underwent 8-plate guided growth knee surgery. Their mother (II:1) has not been clinically evaluated, but she reportedly has a history of chronic mild hip joint pain. The maternal grandmother (I:2) has experienced hip joint pain since childhood. At age 35, she underwent bilateral hip replacement surgery, and her right hip has since undergone three revision surgeries. She also had surgical procedures on her right knee, however, there are no records available regarding the procedure that was performed. Although she is currently pain-free, she walks with a gait. An older maternal half-brother (III:1), aged 18, also reportedly has hip pain and walks with a limp. The siblings were heterozygous for a splice variant in *RPL13*, NM_000977.3: c.477+1G>A. Confirmatory molecular testing of the mother, grandmother, and maternal half-brother is currently ongoing.

### Molecular findings

Two variants in *RPL13* (NM_000977.3) were identified, c.548G>A, p.(Arg183His), and c.569G>T, p.(Arg190Leu). These variants were detected in two unrelated families and segregated with the phenotype of SEMD-RPL13. None of the variants were present in gnomAD population database or in our in-house databases of 1455 exomes^24^ and 6781 exomes and genomes (Karolinska University Hospital Stockholm). One previously described variant, occurring at the same highly evolutionary conserved position 183, p.(Arg183Pro), has been reported in an individual with SEMD-RPL13^6^. Based on the ACMG-AMP guidelines, we consider the two variants likely pathogenic. Three patients, including two half-siblings from family 7, were confirmed heterozygous for the previously reported splice variant c.477+1G>A, classified as pathogenic. The father in family 6 carries the variant in 29% of his alleles in peripheral blood DNA, making him a mosaic carrier for the genetic alteration. Functional studies by Le Caignec et al. demonstrated that this variant induces an aberrant mRNA, containing 54 bp of intron 5, resulting in the insertion of 18 amino acids within the protein^6^.

### Predicted structural and functional effects of the eL13 variants

All known eL13 missense mutations are found in H7, as shown in Fig. 1c, d. The residues in H7, except for Arg190, are highly evolutionary conserved with nearly maximal ConSurf-DB scores (8 out of 9)^25^ (Figure S1). H7 is amphipathic and has two distinct “faces”, one being primarily apolar/hydrophobic and interacts with 60S ribosomal proteins (eL27a and eL36), and the other polar/hydrophilic containing positively charged arginine residues (Arg183, Arg186, and Arg190), which bind to negatively charged phosphates (nucleotides G979, G980, and U981) in a 10-mer hairpin structure (AUGAAGGUGA) from ES9L 28S rRNA expansion segment. While interactions with eL27a and eL36 are primarily hydrophobic and involve H7 apolar residues (Ala178, Ala185), the mutated Arg183 and Arg190 are side by side, on the opposite surface of H7, and come in contact with phosphate groups, resembling “arginine forks”, previously described by Chavali et al.^26^. Notably, the recognized 28S rRNA segment forms a double-stranded RNA hairpin stabilized by a non-canonical G·U wobble pair configuration (U975-G980), which is known to mediate highly specific protein-RNA recognition^27^. This hairpin structure is further recognized by Arg190 binding to unpaired G979, and a distinct bend is present in helix 7, allowing interactions between the G·U wobble pairs and the protein, as previously described^28^. All currently known variants are in a 30 Å sphere where eL13 interacts with 28S rRNA through two specific RNA-binding sequences, as seen in Fig. 1 and Figure S1C. These amino acid substitutions can potentially disrupt the interaction of eL13 with 28S rRNA and the neighboring ribosomal protein through different mechanisms: (1) Arg183His introduces a shorter and bulkier side-chain with pH-variable charge; (2) Arg190Leu removes the positive charge of H7 that stabilizes RNA binding; (3) Arg183Pro introduces a kink changing the orientation of H7, and (4) Ala178Glu introduces negative charges that would repel the negatively charged RNA. In contrast to these missense mutations, the insertion of 18 amino acids after Asn159, previously reported by le Caignec et al, located approximately 30 Å away, targets the non-conserved extended loop that weakly binds ES7L 28S rRNA (nucleotide A509). This extended loop acts as a physical spacer between the N- and C-terminal RNA-binding regions of eL13. Therefore, this insertion would also disrupt the proteins’ interaction with rRNA within the missense cluster by pushing H7 further away.

## Discussion

Spondyloepimetaphyseal dysplasia with severe short stature, *RPL13* related (SEMD-RPL13) is a recently identified condition caused by heterozygous variants in a ribosomal protein, eL13. Previous reports have identified 13 individuals from nine families with six different disease-causing variants in *RPL13*^6,16,17^. This study reports two previously unreported variants in *RPL13* and provides phenotypic and radiological data from 12 additional individuals from seven unrelated families. We compare our patients’ clinical and molecular profiles alongside those previous reported individuals with SEMD-RPL13 (Tables S4, S5). We conclude that the most common features of SEMD-RPL13, were mild spondylar dysplasia, lower limb epimetaphyseal dysplasia, and delayed carpal bone ossification. In our study, height ranged from −5,8 to −1,2 SDS and the clinical manifestations varied greatly (Table 1, Table S2), consistent with previous reports of SEMD-RPL13 and other ribosomopathies^5,6,16,17,29,30^. Family 1 had a reportedly unaffected individual (1-I:3) with the c.548G>A variant in *RPL13*. However, this individual was not available for clinical evaluation, and mild skeletal involvement cannot be excluded. None of the patients presented with any hematological or immunological manifestations, consistent with previous reports of SEMD-RPL13^6^. *RPL13* comprises six exons (Fig. 1f) and encodes a 211 amino acid protein, eL13. Previous studies by Le Caignec et al.^6^ and Costantini et al.^15^ concurred that SEMD-RPL13 mutations impair the ribosomal function of eL13 by disrupting the interactions with 28S rRNA and adjacent proteins eL36 and eL33. Le Caignec et al. showed that siRNA mediated knockdown of RPL13 in HeLa cells caused abrogation of pre-rRNA processing. However, this was not observed in the patients’ fibroblasts^6^, and a zebrafish knockout model only exhibited a partial manifestation of the phenotype^16^. In yeast, a decrease in eL13 at an early stage of ribosomal assembly results in ribosomal maturation arrest^31,32^. The function of eL13 and the molecular mechanisms underlying ribosome dysfunction in humans are still inadequately understood, creating an intriguing research question that has yet to be addressed. Several potential mechanisms have been proposed to explain the phenomenon^33,34^. The tissue-specific skeletal manifestations suggest that eL13 is particularly significant for endochondral ossification and bone growth. However, this alone cannot fully explain the significant clustering of SEMD-RPL13 mutations in the H7 domain or the specific skeletal phenotype caused by these variants. We could not find any heterozygous or homozygous truncating variants in the gene or deletions involving *RPL13* in Decipher (https://www.deciphergenomics.org/browser), gnomAD (https://gnomad.broadinstitute.org), or local in-house databases. Therefore, it is possible that the disease mechanism of SEMD-RPL13 is not caused by a complete loss-of-function or haploinsufficiency, since carrier status of heterozygous deletions has not been reported in humans and could potentially be incompatible with life. Our analysis of the known pathogenic eL13 variants has revealed that they are located within a 30 Å sphere, where eL13 interacts with 28S rRNA through two specific RNA-binding sequences (Fig. 1 and Figure S1). One of these RNA binding sequences is located at the non-conserved extended loop, which weakly binds to ES7L of the 28S rRNA. This loop comprises the previously reported splice variant, resulting in an 18 amino acid insertion into eL13. The other RNA binding sequence is in the H7 arginine-rich RNA binding motif, where the missense variants are clustered. The phenotypic similarity of the insertion and the clustered missense variants suggests that they may be functionally associated in binding rRNA motifs with precise spacing and highly specific recognition properties, primarily through arginine-mediated RNA recognition by the arginine fork and G·U-wobble base pair interaction. Growing evidence indicates that ribosomal proteins have essential functions beyond their role as structural components of the ribosome, such as modulating the translation of specific mRNAs by binding to their 5’- and 3’ UTRs^21,22^. Interestingly, it has been demonstrated that eL13 directly binds to the 3’UTR mRNA of NF-κB1, forming a complex with Rig-I^23^. The mRNA of NF-κB1 contains short sequences resembling that of the 28S rRNA hairpin recognized in ribosomal structures. NF-κB1 is a crucial regulator of the skeletal system, where it controls the differentiation or activity of major skeletal cell types and receptors, including IGF1 and BMP2^35^. Furthermore, a previous study has shown that eL13 can favor the induction and activation of the NF-κB1 promoter^36^. Overall, based on our 3D structural analysis of the eL13 variants, we propose that the skeletal phenotype of SEMD-RPL13 may be due to the disruption of the extra-ribosomal binding of eL13 to NF-κB1 mRNA. Further molecular studies are required to verify this proposed disease mechanism and to decipher these puzzling tissue-specific effects of eL13 mutations.

## Materials and methods

### Subjects

A detailed health history, clinical photographs, and radiographs were obtained after receiving written informed consent from the affected individuals, their participating family members and the parents/legal guardians of the participating children. Written informed consent for publication of clinical photographs was obtained from the patient/legal guardians. The study received ethical approval from Institutional Ethics Committee, Kasturba Medical College and Hospital, Manipal (IEC:921/2018), Baylor College of Medicine Institutional Review Board (H-25722), Stellenbosch University Faculty of Medicine and Health Sciences HREC (Undiagnosed Disease Programme; N18/03/031) and by Regional Ethical Review Board, Stockholm (protocol numbers 2014/983-31/1 and 2012/2106-31/4 (ClinicalTrials.gov Identifier: NCT05876416). The individual from Norway was diagnosed in a clinical setting and written informed consent was obtained for the publication of clinical and genetic data. We calculated the SDS of patients’ length/height using WHO Child growth standards (https://www.who.int/toolkits/child-growth-standards/standards), with values of 18 years for adult participants.

### Massive parallel sequencing and variant analysis

Various modifications of massive parallel sequencing (MPS) were used in this study, which are detailed in Supplemental Table 1. Exome sequencing and variant calling were performed as previously described^37,38^. For patient 3, a standard bioinformatic approach for base calling (RTA), read alignment (BWA) and variant detection (Best practice variant calling GATK) were used^39–41^. Clinical whole genome sequencing (WGS) was conducted on DNA samples from seven individuals. All called and annotated variants were assigned a prioritization score using a rank model and analyzed in Scout, an interface for variant analysis, as previously described^42^. Variants were classified according to the guidelines and criteria of the American College of Medical Genetics and Genomics and Association of Molecular Pathologists (ACMG-AMP)^43^. Validation and segregation analysis of the variants were performed using Sanger sequencing for all patients included in the study, except for patient 3. The variants identified in this study have been submitted to the ClinVar database (accession numbers: SCV001984874, SCV002549921, SCV002549055, SCV002549920, SCV003935993, and SCV002058197).

### Structural mapping and analyses

Structures for human eL13 (Uniprot code: P26373) were obtained from the Protein Data Bank (PDB: www.wwpdb.org^8^, all of them belonging to ribosomal subunits and their assemblies onto ribosomes and disomes. All 37 entries were examined for the general conformation and interactions of eL13. The representative entry 6OLG^44^, which has a resolution of 3.4 Å, was used for further mutational mapping and interpretation. Distances between RNA and other proteins were calculated, and images were generated with the visualization software VMD (Visual Molecular Dynamics)^45^. Conservation scores were retrieved from the ConSurf Database (https://consurf.tau.ac.il/)^25^.

### Reporting summary

Further information on research design is available in the Nature Research Reporting Summary linked to this article.

### Supplementary information

REPORTING SUMMARY

Supplementary Information

## Data Availability

According to European law (https://eur-lex.europa.eu/eli/reg/2016/679/oj), the General Data Protection Regulation (GDPR) prohibits the sharing of entire genome sequencing datasets from the European patients. In addition, the dataset for this article is not publicly available due to concerns regarding participant/patient anonymity, since sharing of raw or modified genome sequencing dataset is not included in our ethical permit or informed consent signed by the patients. Anonymized clinical data is available by contacting the corresponding authors and will be provided within two weeks from the request. All variants identified in this study have been submitted to the ClinVar database (accession numbers SCV001984874, SCV002549921, SCV002549055, SCV002549920, SCV003935993, and SCV002058197). We are prepared to share small subsets of variants of interest upon a reasonable request to the corresponding authors.
