# Supplementary material for: Clinical, genetic and structural delineation of RPL13-related spondyloepimetaphyseal dysplasia suggest extra-ribosomal functions of eL13
Source: NPJ Genom Med. 2023 Nov 22;8:39. doi: 10.1038/s41525-023-00380-x (PMC10665555; doi:10.1038/s41525-023-00380-x)
Supplement: Supplementary file 1 — REPORTING SUMMARY [file 41525_2023_380_MOESM1_ESM.pdf]

nature portfolio

Corresponding author(s): Grigelioniene

Last updated by author(s): Aug 2, 2023

Reporting Summary

Nature Portfolio wishes to improve the reproducibility of the work that we publish. This form provides structure for consistency and transparency in reporting. For further information on Nature Portfolio policies, see our Editorial Policies and the Editorial Policy Checklist.

Statistics

For all statistical analyses, confirm that the following items are present in the figure legend, table legend, main text, or Methods section.

n/a	Confirmed	
☒	☐	The exact sample size (*n*) for each experimental group/condition, given as a discrete number and unit of measurement
☒	☐	A statement on whether measurements were taken from distinct samples or whether the same sample was measured repeatedly
☒	☐	The statistical test(s) used AND whether they are one- or two-sided<br>*Only common tests should be described solely by name; describe more complex techniques in the Methods section.*
☒	☐	A description of all covariates tested
☒	☐	A description of any assumptions or corrections, such as tests of normality and adjustment for multiple comparisons
☒	☐	A full description of the statistical parameters including central tendency (e.g. means) or other basic estimates (e.g. regression coefficient) AND variation (e.g. standard deviation) or associated estimates of uncertainty (e.g. confidence intervals)
☒	☐	For null hypothesis testing, the test statistic (e.g. *F*, *t*, *r*) with confidence intervals, effect sizes, degrees of freedom and *P* value noted<br>*Give P values as exact values whenever suitable.*
☒	☐	For Bayesian analysis, information on the choice of priors and Markov chain Monte Carlo settings
☒	☐	For hierarchical and complex designs, identification of the appropriate level for tests and full reporting of outcomes
☒	☐	Estimates of effect sizes (e.g. Cohen's *d*, Pearson's *r*), indicating how they were calculated

Our web collection on statistics for biologists contains articles on many of the points above.

Software and code

Policy information about availability of computer code

Data collection	None.
Data analysis	The algorithms used for MPS analyses are previously published and referred to in the article. Software that was used in this study was SCOUT, https://github.com/Clinical-Genomics/scout

For manuscripts utilizing custom algorithms or software that are central to the research but not yet described in published literature, software must be made available to editors and reviewers. We strongly encourage code deposition in a community repository (e.g. GitHub). See the Nature Portfolio guidelines for submitting code & software for further information.

Data

Policy information about availability of data

All manuscripts must include a data availability statement. This statement should provide the following information, where applicable:

- Accession codes, unique identifiers, or web links for publicly available datasets
- A description of any restrictions on data availability
- For clinical datasets or third party data, please ensure that the statement adheres to our policy

According to European law (https://eur-lex.europa.eu/eli/reg/2016/679/oj) the General Data Protection, the raw data from the European patients cannot be shared as a whole data set. Nevertheless, we can share small subsets of variants of interest upon a reasonable request. The dataset for this article is not publicly available due to concerns regarding participant/patient anonymity. Requests to access the dataset should be directed to the corresponding author.

Research involving human participants, their data, or biological material

Policy information about studies with human participants or human data. See also policy information about sex, gender (identity/presentation), and sexual orientation and race, ethnicity and racism.

Reporting on sex and gender	Patients biological sex is reported in the clinical summaries with their consent. We did not collect any data regarding their gender/presentation.
Reporting on race, ethnicity, or other socially relevant groupings	We report patients ethnicity since it is relevant for genetic analyses, but not evaluate socioeconomic status of the participants.
Population characteristics	Population characteristics are reported in the manuscript (relevant diagnoses) with the consent from the study participants.
Recruitment	Participants were selected according to their molecular genetic diagnose.
Ethics oversight	Institutional Ethics Committee, Kasturba Medical College and Hospital, Manipal (IEC:921/2018), Baylor College of Medicine Institutional Review Board (H-25722), Stellenbosch University Faculty of Medicine and Health Sciences HREC (Undiagnosed Disease Programme; N18/03/031) and by Regional Ethical Review Board, Stockholm (014/983-31/1 and 2012/2106-31/4

Note that full information on the approval of the study protocol must also be provided in the manuscript.

Field-specific reporting

Please select the one below that is the best fit for your research. If you are not sure, read the appropriate sections before making your selection.

☒ Life sciences ☐ Behavioural & social sciences ☐ Ecological, evolutionary & environmental sciences

For a reference copy of the document with all sections, see nature.com/documents/nr-reporting-summary-flat.pdf

Life sciences study design

All studies must disclose on these points even when the disclosure is negative.

Sample size	Not relevant we collected individuals with RPL13 related skeletal dysplasia, which is ultra-rare diagnosis.
Data exclusions	no data was excluded.
Replication	The genetic findings were confirmed using two different sequencing method, when necessary.
Randomization	Not relevant for this study since we did compare any groups of the patients.
Blinding	Not relevant see above.

Reporting for specific materials, systems and methods

We require information from authors about some types of materials, experimental systems and methods used in many studies. Here, indicate whether each material, system or method listed is relevant to your study. If you are not sure if a list item applies to your research, read the appropriate section before selecting a response.

Materials & experimental systems

n/a	Involved in the study	
☒	☐	Antibodies
☒	☐	Eukaryotic cell lines
☒	☐	Palaeontology and archaeology
☒	☐	Animals and other organisms
☐	☒	Clinical data
☒	☐	Dual use research of concern
☒	☐	Plants

Methods

n/a	Involved in the study	
☒	☐	ChIP-seq
☒	☐	Flow cytometry
☒	☐	MRI-based neuroimaging

Clinical data

Policy information about clinical studies

All manuscripts should comply with the ICMJE guidelines for publication of clinical research and a completed CONSORT checklist must be included with all submissions.

Clinical trial registration	NCT05876416
Study protocol	Karolinska Institutet Protocol Record 910715, also see the Ethics oversight.
Data collection	Karolinska Institutet and Karolinska University Hospital, Stockholm, Sweden, Kasturba Medical College and Hospital, India, Baylor College of Medicine, Baylor, USA, Stellenbosch University Faculty of Medicine, South Africa, Oslo University Hospital, Oslo, Norway. 2021-2022.
Outcomes	Increased knowledge on molecular causes and clinical course of RPL13 related skeletal dysplasia.
