# Supplementary material for: Clinical, genetic and structural delineation of RPL13-related spondyloepimetaphyseal dysplasia suggest extra-ribosomal functions of eL13
Source: NPJ Genom Med. 2023 Nov 22;8:39. doi: 10.1038/s41525-023-00380-x (PMC10665555; doi:10.1038/s41525-023-00380-x)
Supplement: Supplementary file 2 — Supplementary Information [file 41525_2023_380_MOESM2_ESM.pdf]

Supplementary Tables

Supplemental Table 1. Sequencing methods used in this study						
**Individual**	**Sequencing method**	**Platform**	**Library Prep**	**Gene panel**	**Validation**	**Centra**
**1-II:7**	WES	Illumina sequencing platform	Nextera Rapid Capture Exomes library kit	-	Sanger	Kasturba Medical College and Hospital
**1-III:3**	WES	Illumina sequencing platform	SureSelect Human All Exon V2 (CREV2)	-	Sanger	Kasturba Medical College and Hospital
**2-II:2**	WES	Illumina sequencing platform	SureSelect Clinical Research Exome v3 (CREV3)	-	Sanger	Kasturba Medical College and Hospital
**3**	WES	NextSeq500	Human Core Exome with RefSeq spike in (Twist Bioscience)	Skeletal dysplasia gene list*	-	Telemark Hospital Trust
**4-I:1**	WGS	NovaSeq 6000	TruSeq DNA PCR Free	-	NA	Karolinska University Hospital
**4-I:2**	WGS	NovaSeq 6000	AmpFREE Low DNA Library Kit (Lucigen)	-	NA	Karolinska University Hospital
**4-II:1**	WGS	NovaSeq 6000	TruSeq DNA PCR Free	Skeletal dysplasia gene list*	Sanger	Karolinska University Hospital
**4-II:4**	WGS	NovaSeq 6000	TruSeq DNA PCR Free	Skeletal dysplasia gene list*	Sanger	Karolinska University Hospital
**5-II:2**	WGS	HiSeq X	AmpFREE Low DNA Library Kit (Lucigen)	Skeletal dysplasia gene list*	Sanger	Karolinska University Hospital
**5-III:1**	WGS	NovaSeq 6000	TruSeq DNA PCR Free	Skeletal dysplasia gene list*	Sanger	Karolinska University Hospital
**6-II:1**	WGS	NovaSeq 6000	TruSeq DNA PCR Free	Skeletal dysplasia gene list*	Sanger	Karolinska University Hospital
**6-I:1**	WES	NovaSeq 6000	Twist Biosciences core exome bait set and Kapa Hyper Plus	-	Sanger	Karolinska University Hospital
**7-III:2**	WES	NovaSeq 6000	TruSeq DNA PCR Free	-	Sanger	3billion Inc
**7-III:3**	WES	NovaSeq 6000	TruSeq DNA PCR Free	-	Sanger	3billion Inc
*Skeletal dysplasia gene list corresponding to Genomics England PanelApp, https://panelapp.genomicsengland.co.uk/panels/309/; NA, not applicable.						

Supplemental Table 2. Summary of clinical information of the patients with SEMD-RPL13 in this study

	Present study											
**Family**	**1**			**2**	**3**	**4**		**5**		**6**	**7**	
**Individual**	III:4	II:6	II:5	II:2	3	II:1	II:4	III:1	II:2	II:1	III:2	III:3
**Age (years)**	3	41	35	9	20	31	24	25	64	6.5	6	2
**Sex**	M	M	M	M	F	F	M	F	F	M	M	F
**Clinical features**												
**Facial dysmorphism**	-	-	-	-	Mild epicanthal folds	-	-	-	-	-	-	-
**Height cm (SDS#)**	83 (-3.5)[b]	140 (-5.0)[c]	134 (-5.8)[c]	129 (-0.59)[d]	137.5 (-3.9)[c]	155 (-1.2)[c]	155.8 (-2.5)[c]	142.8 (-3.1)[c]	155.6 (-1.2)[c]	105.8 (-2.5)	100 (-3.2)	76 (-3.2)
**Birth length at term cm (SDS[a])**	NA	NA	NA	NA	NA	Normal[e]	Normal[e]	51 (1.0)	Normal	51 (0.6)	45 (-2.6)	44 (-2.8)
**Birth weight at term kg (SDS[a])**	2,5 (-1.9)[e]	NA	NA	2.9 (-1.0)[e]	NA	NA	NA	4.0 (1.6)	NA	3.5 (0.4)	NA	NA
**Chest anomalies**	Mild *pectus carinatum*	-	-	Mild *pectus excavatum*	NA	-	*Pectus excavatum*	Mild *pectus excavatum*	-	-	-	-
Genu varum	+	+	+, unilateral	-	+	-	-	+	+	-	-	+
**Joint hyperlaxity**	+	+	+	-	-	-	-	-	-	+	+	+
**Other**	-	-	-	-	-	Patellar luxation	Myopia	Patellar luxation,GH treated	Rectal cancer, degenerative joint disease	*Genu valgum (mild), pes planus*	*Genu valgum, pes planus*	*Pes planus*
**Molecular details**												
***RPL13* Variant NM_000977.3**	c.548G>A	c.548G>A	c.548G>A	c.548G>A	c.569G>T	c.569G>T	c.569G>T	c.548G>A	c.548G>A	c.477+1G>A	c.477+1G>A	c.477+1G>A
**Protein change**	p.(Arg183 His)	p.(Arg183H is)	p.(Arg183Hi s)	p.(Arg183H is)	p.(Arg190Leu)	p.(Arg190L eu)	p.(Arg190L eu)	p.(Arg183His)	p.(Arg183His)	p.(Asn159Val 160ins18)	p.(Asn159Val 160ins18)	p.(Asn159Val160 ins18)
**Location**	Exon 6	Exon 6	Exon 6	Exon 6	Exon 6	Exon 6	Exon 6	Exon 6	Exon 6	Intron 5	Intron 5	Intron 5
**ACMG Classification**	4	4	4	4	4	4	4	4	4	5	5	5

+, feature present; -, feature absent; NA, data not available; M, male; F, female; [a] Patient height and weight SDS was calculated using WHO growth references (http://www.who.int/childgrowth/standards/en/); [b] At 3 years, [c] Adult height, [d] At 9 years, [e] Gestational age unknown but said to be normal

Supplemental Table 3. Summary of radiographic features in this study with SEMD-RPL13

	Present study									
**Family**	**1**	**2**	**3**	**4**		**5**		**6**	**7**	
**Individual**	III:4	II:2	3	4-II:1	II:4	III:1	II:2	II:1	III:2	III:3
**Age at radiographic evaluation (years)**	3	9	11	18	15	7	42	7	6	2
**Sex**	M	M	F	F	M	F	F	M	M	F
**Upper extremities**										
**Carpal bone ossification**	Unossified[a]	Delayed[b]	+[c,f]	NA	NA	Delayed[b, d]	NA	Normal	Delayed[b,h]	Delayed
**Epimetaphyseal changes of the hand and wrist**	-	-	-	NA	NA[i]	-	NA	-	-	-
**Spine**										
**Scoliosis**	-	-	-	-	+, mild	-	-	-	-	-
**Lordosis**	-	-	+[g]	+, mild	-	+, severe	-	-	-	+
**Kyphosis**	-	+, thoracic	+, thoracic[g]	-	-	-	-	-	-	-
**Platyspondyly**	+, mild	+, mild	+, mild[g]	+, mild	-	+, mild	-	+	-	-
**Irregular vertebral end plates**	-	+	+[g]	+	+	+	+	+	+	+
**Hips and lower extremities**										
Coxa vara	+	+	+, severe	+, mild	+, mild	+	+, mild	+, mild	-	-[k]
**Bowed femora**	-	-	+, as a young child	-	-	-	-	-	+	+
**Capital femoral epiphyses**	Unossified	Flat and irregular	Flat and irregular	Mildly flat	Severely flat and irregular	Flat, severely[e]	Flat	Flat	Flat and delayed	Unossified
**Short femoral necks**	+	+	+	+	+	+	+	+	+	+
**Metaphyseal irregularities of the proximal femora**	+	+	+	+	-[h]	+	-[h]	+	+, severe	+, severe
**Epiphyseal changes of the knees**	+, small epiphyses	NA	+, defective ossification of the medial aspect of the proximal tibia	+, mild	+, mild	+, small epiphyses	Degenerative joint disease	-	+, mildly flat epiphyses	+, mildly flat
**Metaphyseal changes of the knees**	+, irregular	NA	+[j]	-	+, mild	+, irregular	-	+	+, mild	+, mild
**Epiphyseal changes of the distal tibia and fibula**	+, small epiphyses	NA	NA	NA	NA	+, small epiphyses[a]	NA	-	-	-
**Metaphyseal irregularities of the distal tibia and fibula**	+	NA	NA	NA	NA	+[e]	NA	+	-	-
**Other**			High rising greater trochanters, shallow acetabula		High rising greater trochanters	Short neural arches of lower lumbar spine, spinal canal stenosis	High rising greater trochanters, narrow joint spaces of the hip			

+, feature is present; -, feature is absent; NA, data not available; AVN, avascular necrosis; [a] At 3 years; [b] Particularly of proximal row; [c] Hypoplastic lunate and scaphoid; [d] At 5 years, [e] At 10 years, [f] At 16 years, [g] At 15 years; h, growth plate is closed and metaphyseal changes could be subsided; [i] No epimetaphyseal changes in upper arm; j, mild, proximal tibia only, [h] At 8 years. [i] The vertebral bodies show central notches and anterior ossification defects. [j] At 4 years, [k] Difficult to determine due to unossified femoral heads

Supplemental Table 4. Summary of the literature reports regarding clinical information of previously described individuals with SEMD-RPL13												
	**Le Caignec et al.,2015**				**Reinsch et al.,2020**	**Costantini et al., 2021**						
**Family**	**6**	**7**	**8**	**9**	**10**	**11**	**12**				**13**	**14**
**Age (years)**	4	3	4	2	9	4.5	4.5	23	49	25	9.5	3
**Sex**	M	M	M	M	M	M	F	F	F	F	M	M
**Consanguinity**	-	-	-	-	NA	-	-	-	-	-	-	-
**Origin**	NA	NA	NA	NA	NA	Finnish	Korean	Korean	Korean	Korean	Korean	French/ Congolese
**Growth**												
**Height cm (SDS)**	severe short stature	severe short stature	severe short stature	severe short stature	129 (-0.59)	69 (-8.5)	85 (-4.7)	144.5 (-3.5)	135.8 (-5.6)	157.9 (-0.6)	-90.5 (7.2)	53 (-2.45[b] -6.0[c])
**Birth length at term cm (SDS[a])**	49 (-0.5)	50 (0.1)	Normal	Normal	51 (0.6)	42 (-4.2)	NA	NA	NA	NA	NA	NA
**Birth weight at term kg (SDS[a])**	3.4 (+0.1)	4.0 (+1.3)	NA (>2)	NA (>2)	3.69 (0.7)	2.45 (-2.9)	NA	NA	NA	NA	NA	NA
**Clinical features**												
**Facial dysmorphism**	NA	NA	NA	NA	NA	+, mild coarsness, low nasal bridge	+,mild	NA	NA	NA	-	NA
**Chest anomalies**	NA	NA	NA	NA	-	Narrow thorax	Narrow thorax	*Pectus excavatum*	-	-	*Pectus excavatum*	Narrow thorax
Genu varum	+	+	+	+	-	NA	+	-[d]	-	-	+	+
**Joint hyperlaxity**	-	-	-	-	-	NA	NA	NA	NA	-	+	NA
**Other**	-	-	-	-	-	hypoplastic primary teeth with hypomineralization	-		*Genua valga*		Subglottic stenosis	Deceased at 3y respiratory insufficiency
**Molecular details**												
***RPL13* Variant NM_000977.3**	c.477+1G>T	c.477+2T>C	c.477+1G>A	c.548G>C	c.477+1G>A	c.533C>A	c.533C>A	c.533C>A	c.533C>A	c.533C>A	c.553G>C	c.477+1G>T
**Protein change**	p.(Asn159_Val160ins18)	p.(Asn159_Val160ins18)	p.(Asn159_Val160ins18)	p.(Arg183Pro)	p.(Asn159_Val160ins18)	p.(Ala178Glu)	p.(Ala178Glu)	p.(Ala178Glu)	p.(Ala178Glu)	p.(Ala178Glu)	p.(Ala185Pro)	p.(Asn159_Val160ins18)
**Location**	Intron 5	Intron 5	Intron 5	Exon 6	Intron 5	Exon 6	Exon 6	Exon 6	Exon 6	Exon 6	Exon 6	Intron 5
**ACMG Classification**	5	5	5	5	5	5	5	5	5	5	5	5
+, feature is present; -, feature is absent; NA, data not available; M, male; F, female; y, years; m, months; [a] Patient height and weight SDS was recalculated using original data from the references and WHO growth references (http://www.who.int/childgrowth/standards/en/); [b]At 2 months, [c]At 14 months, [d]*Genu valgum*												

**Supplemental Table 5. Summary of the radiographic features in previously reported individuals with SEMD-RPL13**												
	**Le Caignec et al.,2015**				**Reinsch et al.,2020**	**Constantini et al., 2021**						
**Family**	**6**	**7**	**8**	**9**	**10**	**11**	**12**				**13**	**14**
**Age (years)[a]**	4	3	4	2	9	4	4	23	49	25	9	3
**Sex**	M	M	M	M	M	M	F	F	F	F	M	M
**Upper extremities**												
**Carpal bone ossification**	NA	NA	NA	NA	NA	Delayed	Delayed	Normal[b]	Normal[b]	Normal[b]	Delayed	NA
**Epiphyseal changes**	-	-	+	-	-	+[c]	NA	-	-	-	NA	NA
**Spine**												
**Scoliosis**	-	-	-	-	-	+	+	+, mild	-	-	+	-
**Lordosis**	NA	NA	NA	NA	NA	-	-	-	-	-	+	+
**Kyphosis**	NA	NA	NA	NA	NA	NA	NA	NA	NA	-	NA	NA
**Platyspondyly**	+	+	+	+	-	+	+, mild	-	-	-	+	+
**Irregular vertebral end plates**	NA	NA	NA	NA	-	+	+	+	+	-	+	+
**Hips and lower extremities**												
Coxa vara	+	+	+	+	+	-	NA	+	+	-	+	NA
**Bowed femora**	-	+	+	+	-	+, mild	+, mild	-	-	-	+, mild	+, mild
**Capital femoral epiphyses**	Irregular	Unossified	Unossified	Small and irregular	Irregular	Unossified	Unossified	-	Small	-	Unossified	Unossified
**Metaphyseal changes**	+	+	+	+	+	+	+	-[d]	+	-	+	+
**Epiphyseal changes**	+	+	+	+	+	+	+	-	+	-	+	+
**Other**						Vertebral bodies with double humps	Vertebral bodies with double humps					
+, feature is present; -, feature is absent; NA, data not available; M, male; F, female; a, age at radiological evaluation; b, not assessed in childhood; c, wrist; d, growth plate is closed, and metaphyseal changes could be subsided.												

Supplementary Figures

Supplementary Figure 1

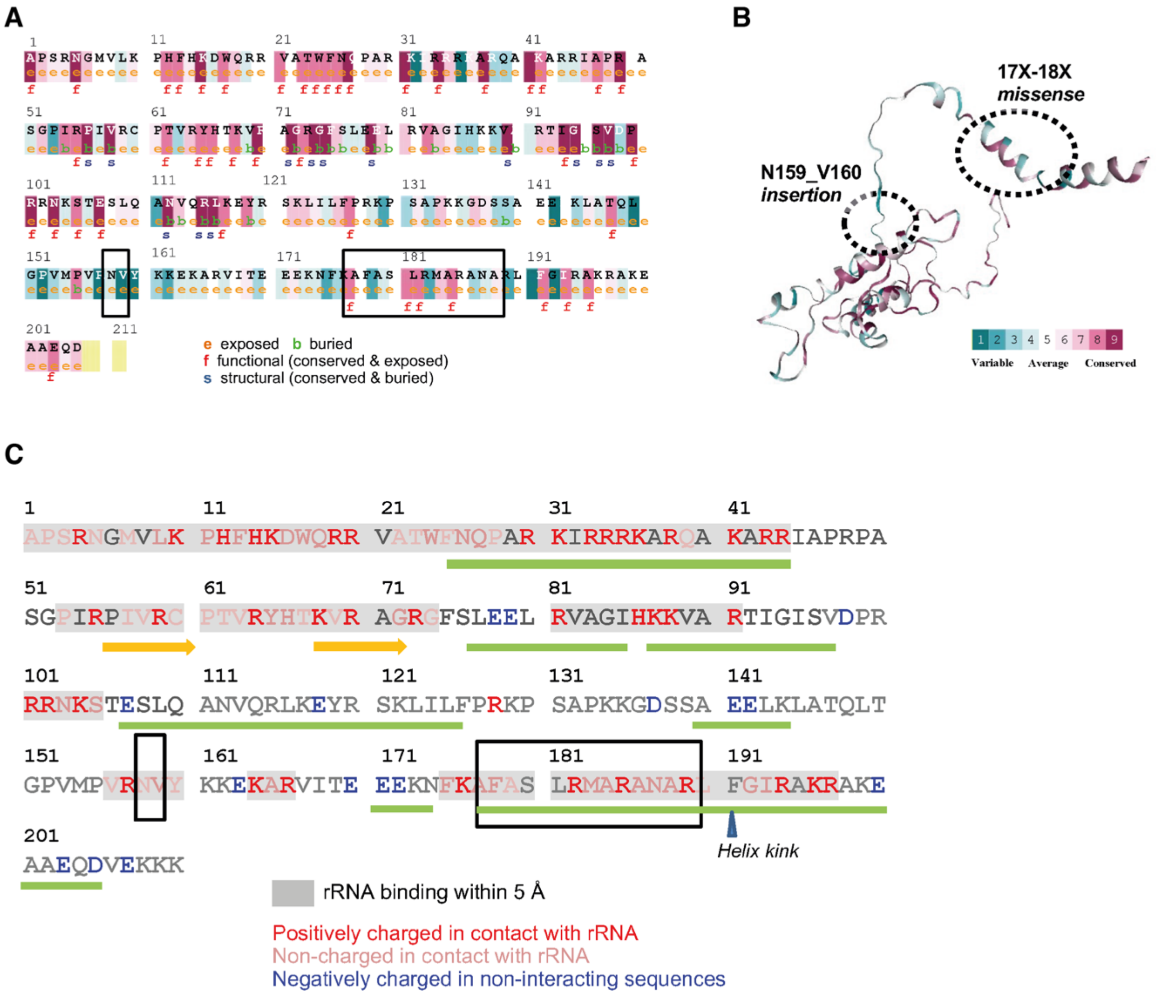

Supplemental Figure 1A. eL13 Residue Conservation Scores obtained from the ConSurf Database (ConSurf-DB https://consurfdb.tau.ac.il/). These scores range from 1 to 9, with 9 indicating maximal evolutionary conservation, and are displayed across the protein's sequence. The eL13 insertion targets the linker region connecting helix 5 and 6 (H5-H6), while the missense mutations cluster on helix 7 (H7), both are highlighted with boxes. Mutations located in conserved and buried regions are expected to disrupt protein folding (indicated in blue as s, for structural) whereas those affecting conserved but exposed residues are anticipated to affect protein conformation and binding to other biomolecules (indicated in red as f, for functional). **B.** ConSurf scores are mapped onto the protein's 3D structure, highlighting the mutation sites. Note that H7, where missense mutations (17X-18X) cluster, is highly conserved (colored pink), while the linker region where the insertion is located is highly variable (colored blue). **C.** RNA binding sites are shown across eL13. Green rectangles represent helices, and orange arrows indicate beta-sheets. The amino acid residues are colored based on their proximity to RNA in the 3D structure. RNA-binding regions are highlighted in grey. eL13 contains two conserved RNA-binding regions separated by a long variable linker, as shown by comparison with ConSurf mapping above (Fig 2A and B). Pathogenic variants in eL13 target the linker region, disrupting the space between RNA-binding regions or cluster at H7 RNA-recognition site.

Supplemental Figure 2

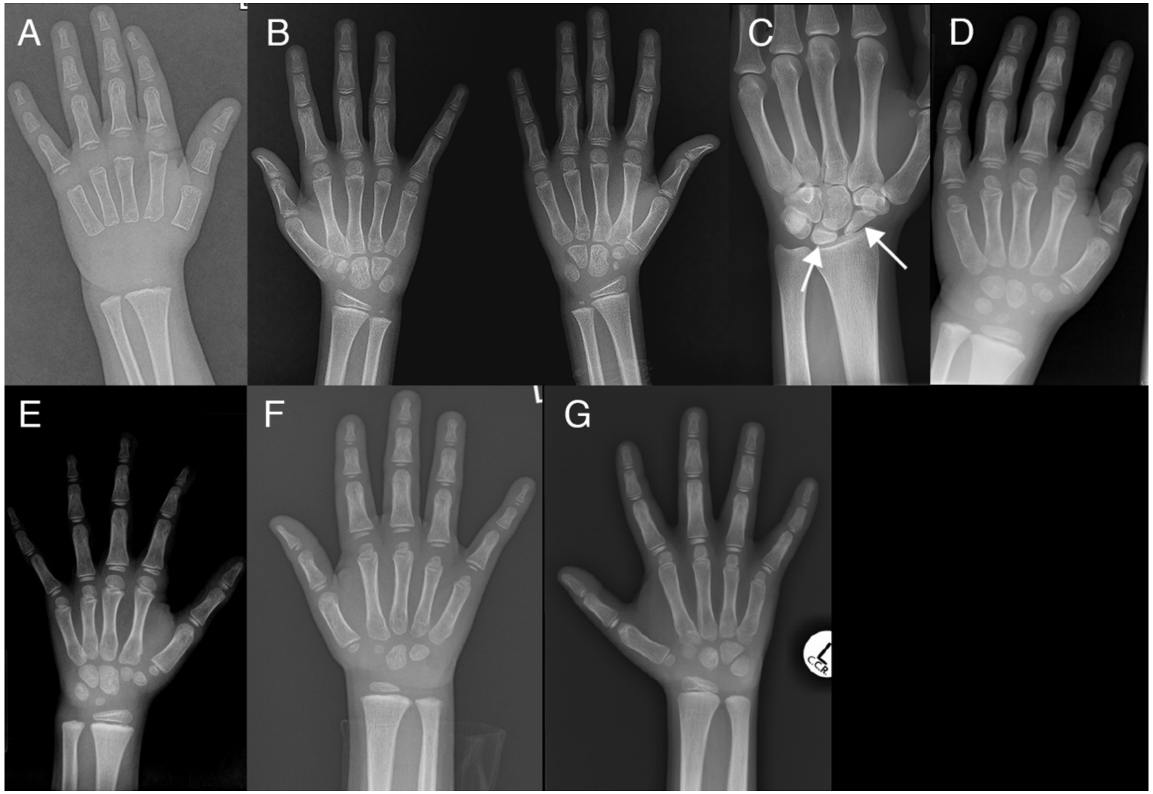

Supplemental Figure 2. Hand radiographs. **A**. Patient 1-III:4, radiograph at age 3 years; showing unossified carpal bones. **B**. Patient 2-II:2, radiograph at age 9 years; showing delayed carpal ossification particularly of the proximal row. **C.** Patient 3, radiograph at age 16 years; showing hypoplastic lunate and scaphoid (white arrows).

D. Patient 5-III:1, radiograph at age 5 years; showing a mildly delayed carpal bone ossification. **E.** Patient 6-II:1, normal radiograph at age 7. **F.** Patient 7-III:2, radiograph at age 8 years and 3 months; showing delayed carpal bone ossification particularly in the radial proximal row. **G.** Patient 7-III:3, radiograph at age 4; showing delayed carpal bone ossification.
